# Evaluation of Anti-Mullerian Hormone Levels, Antral Follicle Counts, and Mean Ovarian Volumes in Chemotherapy-Induced Amenorrhea among Breast Cancer Patients: A Prospective Clinical Study

**DOI:** 10.3390/curroncol30100666

**Published:** 2023-10-19

**Authors:** Çağlar Ünal, Çetin Ordu, Tolga Özmen, Ahmet Serkan İlgun, Filiz Çelebi, Bülent Baysal, Enver Özkurt, Tomris Duymaz, Zeynep Erdoğan İyigün, Sevgi Kurt, Mehmet Alper Öztürk, Kezban Nur Pilancı, Gül Alço, Kanay Yararbaş, Tuba Kayan Tapan, Deniz Can Güven, Gürsel Soybir, Vahit Özmen

**Affiliations:** 1 Division of Medical Oncology, Department of Internal Medicine, Kartal Dr. Lütfi Kırdar City Hospital, Istanbul 34865, Turkey; 2 Division of Medical Oncology, Department of Internal Medicine, Gayrettepe Florence Nightingale Hospital, Istanbul 34349, Turkey; cetinordu@hotmail.com; 3 Division of Gastrointestinal and Oncologic Surgery, Harvard Medical School, Boston, MA 02115, USA; drtolgaozmen@yahoo.com.tr; 4 Division of Gastrointestinal and Oncologic Surgery, Massachusetts General Hospital, Boston, MA 02114, USA; 5 Department of Surgery, Mater Dei Hospital, Msida, MSD 2090, Malta; ilgunserkan@yahoo.com; 6 Department of Radiology, Yeditepe University Hospital, Istanbul 34755, Turkey; elbuken.filiz@gmail.com; 7 Department of Obstetrics and Gynecology, İstanbul Florence Nightingale Hospital, Istanbul 34381, Turkey; baysal.b@gmail.com; 8 Department of General Surgery, İstanbul Florence Nightingale Hospital, Istanbul 34381, Turkey; doctorenver@gmail.com; 9 Department of Physiotherapy and Rehabilitation, Faculty of Health Sciences, Istanbul Bilgi University, Istanbul 34060, Turkey; tomrisduymaz@gmail.com; 10 Department of Physical Theraphy and Rehabilitation, Göztepe Medical Park Hospital, Istanbul 34732, Turkey; znperdogan@gmail.com; 11 Department of Plastic Surgery, İstanbul Florence Nightingale Hospital, Istanbul 34381, Turkey; svgkrt@gmail.com; 12 Department of General Surgery, Biruni Hospital, Istanbul 34295, Turkey; alperozturk79@gmail.com; 13 Division of Medical Oncology, Department of Internal Medicine, Memorial Bahçelievler Hospital, Istanbul 34180, Turkey; kezbannurgunes@gmail.com; 14 Department of Radiation Oncology, Gayrettepe Florence Nightingale Hospital, Istanbul 34349, Turkey; gulalco@gmail.com; 15 Department of Medical Genetics, Demiroglu Bilim University, Istanbul 34394, Turkey; kanayyararbas@gmail.com; 16 Department of Nutrition and Dietetic, Faculty of Health Science, Demiroglu Bilim University, Istanbul 34394, Turkey; kyn.tuba@gmail.com; 17 Division of Medical Oncology, Department of Internal Medicine, Elazıg Fethi Sekin City Hospital, Elazıg 23280, Turkey; denizcguven@hotmail.com; 18 Department of General Surgery, Memorial Şişli Hospital, Istanbul 34384, Turkey; gurselr@yahoo.com; 19 Department of General Surgery, İstanbul School of Medicine, İstanbul University, Istanbul 34093, Turkey; vozmen@istanbul.edu.tr

**Keywords:** anti-mullerian hormone, antral follicle counts, breast cancer, chemotherapy-induced amenorrhea, median ovarian volumes, ovarian reserve

## Abstract

Estradiol (E2), a follicle-stimulating hormone (FSH), AMH, and inhibin B levels, along with AFC and MOV, are used to determine ovarian reserve in pre-menopausal women. Studies have shown that AMH levels are more sensitive than those of E2, FSH, and inhibin B and that AFC and MOV can be used to evaluate ovarian reserve. AMH, AFC, and MOV measurements were performed before and after adjuvant SC in 3-month periods for one year. Patients were classified as experiencing chemotherapy-induced amenorrhea (CIA) if they did not have menstrual cycles for a period of six months or longer following the conclusion of their chemotherapy treatment. We aimed to evaluate the factors affecting chemotherapy-induced amenorrhea in breast cancer patients treated with adjuvant chemotherapy and the performance of baseline measurements of AMH, AFC, and MOV to predict chemotherapy-induced amenorrhea. The effects of different chemotherapy regimens on the AMH level, AFC, and MOV in CIA patients were investigated. Seventy-one patients were eligible for this study, and the median age was 38 years (range: 23–45). The median follow-up was 37 months (range: 20–51), and CIA developed in 62% of the patients. The AMH level and AFC were significantly decreased one year after SC (*p* < 0.0001), whereas MOV was not (*p* = 0.507). AMH levels before chemotherapy (median: 1.520 vs. 0.755, *p* = 0.001) and at the end of the first year (median: 0.073 vs. 0.010, *p* = 0.030) and pre-treatment AFC (median: 12 vs. 4.50, *p* = 0.026) were lower in patients with CIA compared to those without CIA. The AMH levels before SC were the most valuable and earliest factor for predicting CIA development. In addition, there was no difference between the chemotherapy regimens (including or not including taxane) in terms of CIA development.

## 1. Introduction

Cancer significantly contributes to the global disease burden and stands as the second most common cause of mortality in the United States [1]. Breast cancer surpassed lung cancer as the most common cancer with 2.3 million new cases worldwide [2,3,4]. The breast cancer incidence in women under 40 is 7–8%, and in women of reproductive age (under 45 years old), it is 11% [5]. The rate of breast cancer patients in Turkey is much higher in younger populations than it is in Western countries [5]. In a study we conducted with 20,000 breast cancer patients in Turkey, 17.2% were younger than 40, and 37.2% were pre-menopausal [6]. Ovarian function is affected to different extents in patients receiving adjuvant chemotherapy for breast cancer, depending on the type of treatment and age of the patient [7,8,9]. The loss of primordial follicles, reduced ovarian reserve, and ovarian atrophy are observed depending on the treatment [9].

Estradiol (E2), a follicle-stimulating hormone (FSH), AMH, inhibin B levels, antral follicle counts (AFCs), and median ovarian volumes (MOVs) are used to determine the ovarian reserve in pre-menopausal women [10]. In clinical practice, blood tests are frequently used due to their speed, simplicity, and affordability [11,12]. Studies have shown that AMH levels are more sensitive than those of E2, FSH, and inhibin B and that AFC and MOV can be used to evaluate ovarian reserve [13,14].

AMH levels decrease significantly with chemotherapy, according to pre-clinical and retrospective studies [15,16]. Loss of ovarian function in patients receiving chemotherapy is manifested by the discontinuation of menstrual cycles (chemotherapy-induced amenorrhea, CIA) [17]. There are studies that show that different chemotherapy regimens produce amenorrhea at different rates [18]. Cyclophosphamide is the most commonly used and most highly associated with the CIA of the chemotherapy agents in breast cancer adjuvant treatment [7]. Today, docetaxel/cyclophosphamide (TC), less frequently, adriamycin/cyclophosphamide (AC), and combinations are used as adjuvant chemotherapy treatment regimens [19]. It is not entirely clear how much of the incidence of CIA is due to taxane group drugs, which have been widely added to adjuvant chemotherapy in the last two decades [20]. Predicting the effects of both regimens on ovarian reserve may affect treatment choice. However, in some of the patients with CIA, their menstrual cycles return [18]. Currently, with the early diagnosis and modern treatment methods, a longer and healthier life for breast cancer patients preserves their fertility, which is especially important in women who have not given birth and/or desire to give birth [20]. For this purpose, methods such as the freezing of eggs and heterotopic transplantation of partial ovary tissue are the most preferred methods [21].

We aimed to evaluate the factors affecting chemotherapy-induced amenorrhea in breast cancer patients treated with adjuvant chemotherapy and the performance of baseline measurements of AMH, AFC, and MOV to predict chemotherapy-induced amenorrhea.

## 2. Patients and Methods

### 2.1. Study Design

**Figure 1:**
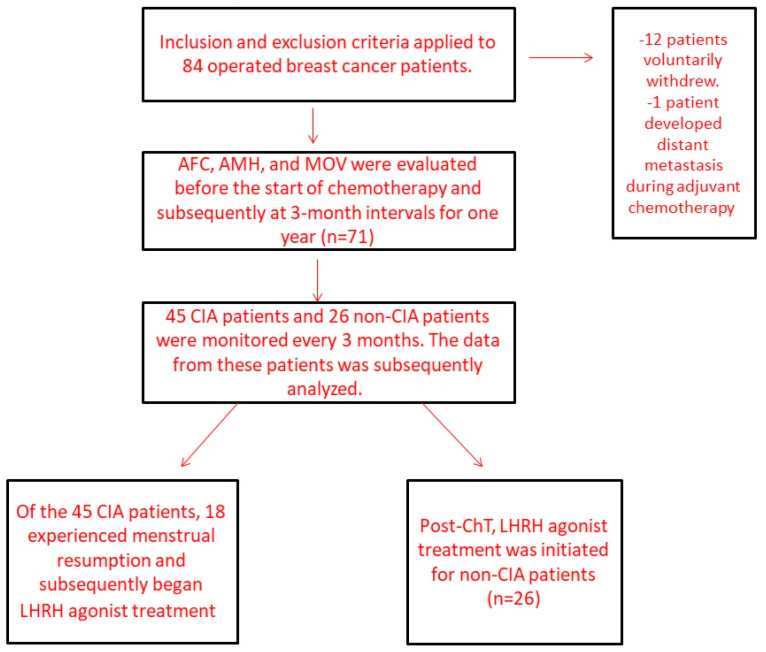
Flow diagram of patients identified and included in the final analysis.

The role of AMH, AFC, and MOV measurements in predicting ovarian reserve was investigated in this prospective clinical study (Figure 1). After obtaining the ethics committee’s approval and ensuring patients decided to undergo adjuvant chemotherapy in the tumor board, informed consent forms were obtained for participation in this study. Serum AMH levels, AFCs, and MOVs of 71 pre-menopausal breast cancer patients were measured before and at 3-month intervals for 1 year after chemotherapy. On the other hand, AFC, AMH, and MOV values measured at the 3-month intervals (second, third, and fourth measurements) were not analyzed because the preliminary results of 31 patients revealed that there were only statistically significant differences between the first and fifth measurements [22]. There were also missing values in the interval measurements.

### 2.2. Patients

The inclusion criteria were as follows:

- Pathologically diagnosed stage I–III breast cancer with complete medical records of menses;
- Perimenopausal status (regular menses at least for one year);
- Undergoing adjuvant chemotherapy based on tumor board’s decision.

The exclusion criteria were as follows:

- Age > 45 years;
- History of other cancer(s);
- Previously underwent chemotherapy;
- Underwent oophorectomy;
- Have a disease causing metabolically abnormalities (renal, hepatic, or cardiac disorders or metabolic disease);
- Individuals who wish or plan to conceive during the post-chemotherapy follow-up period.

Estrogen receptor (ER), progesterone receptor (PR), and human epithelial growth factor receptor 2 (Her-2) levels, histopathology, TNM stage, surgical intervention, and demographic and reproductive characteristics of patients (age, birth-curettage, age at first menstruation, breast-feeding, body mass index (BMI)) were recorded.

### 2.3. Treatment Strategy

The adjuvant chemotherapy was determined by the tumor council; these included docetaxel + cyclophosphamide (TC), adriamycin + cyclophosphamide (AC), or adriamycin + cyclophosphamide (AC) + taxane (T) ± trastuzumab, endocrine treatment (tamoxifen) ± radiotherapy. TC regimen consisted of docetaxel 75 mg/m^2^ and cyclophosphamide 600 mg/m^2^ every 3 weeks for four cycles. And, the AC regimen consisted of doxorubicin 60 mg/m^2^ and cyclophosphamide 600 mg/m^2^ every 3 weeks for four cycles.

The AC + T regimen comprised an initial AC phase, followed by one of two options:

1. Weekly paclitaxel (80 mg/m^2^) was administered for 12 weeks, potentially accompanied by trastuzumab, with the following schedule:

- Initial loading dose: 4 mg/kg; subsequent doses: 2 mg/kg, weekly for 12 weeks, concurrent with paclitaxel.
- Maintenance phase: a single dose of 6 mg/kg trastuzumab every 3 weeks to complete a full year of trastuzumab therapy.

2. Docetaxel (75 mg/m^2^) was administered every 3 weeks over four cycles, potentially accompanied by trastuzumab, with the following schedule:

- Initial loading dose: 8 mg/kg; subsequent doses: 6 mg/kg, every 3 weeks, concurrent with docetaxel.
- Maintenance phase: a single dose of 6 mg/kg trastuzumab every 3 weeks to complete a full year of trastuzumab therapy.

After local and adjuvant treatment (including radiotherapy if needed), patients visited the outpatient clinic every 3 months for the first 3 years of follow-up [23]. CIA was defined as the cessation of menstruation for >6 consecutive months. Resumption of menstruation was defined as regular cyclic bleeding after CIA for >3 months without pathologic etiology [23]. Factors that affect the development of CIA and the resumption of the menstrual cycle were determined. Patients were examined continuously every 3 months during the follow-up period. Luteinizing hormone-releasing hormone (LHRH) agonist use was started in patients who had resumed their menstrual cycle and also in patients who had not developed CIA at the 6-month follow-up after the end of treatment. For LHRH agonist therapy in our study, patients were administered either subcutaneous goserelin (3.6 mg every 28 days) or leuprolide (3.75 mg every 28 days), with the specific dosing and administration frequency determined by our clinical team.

### 2.4. Laboratory Analysis

AMH levels were measured using enzyme-linked immunosorbent assay (ELISA, Helsinki, Finland) using the kit from USCN Life Science, Inc (Buckingham, UK). AFC and MOV measurements were performed by experienced obstetrics, gynecology, and infertility specialists and radiologists using transvaginal ultrasonography (EV9-4 probe, Siemens Acuson S2000, Erlangen, Germany). Ovarian volume data were calculated as the mean and AFC data as the sum for both ovaries. Mean ovarian volume (MOV) was calculated with the use of this formula: (A × B × C × 0.52). AFC was determined as the number of follicles that were 2–10 mm in diameter for both ovaries. AFC, AMH, and MOV values measured at the 3-month intervals (second, third, and fourth measurements) were not analyzed because the preliminary results of 31 patients revealed that there were only statistically significant differences between the first and fifth measurements [22]. On the other hand, there were missing values in the interval measurements. All AMH serum samples were collected, frozen, and run in a single batch to mitigate batch-to-batch assay variability.

### 2.5. Statistical Analysis

The median age, follow-up duration, and minimum–maximum interval of the patients were determined. The mean values and standard deviation for the nominal values were calculated. The differences between the analyzed groups (CIA, resumption of menses, AC vs. TC, chemotherapy with or without taxanes, age of >35 vs. 35 years) were investigated using the chi-squared test. Based on CIA development, the measured AMH, AFC, and MOV values were statistically analyzed using the Mann–Whitney U test. As the values measured at the end and beginning of the first year of treatment were complete, the 2nd, 3rd, and 4th measurements at the 3-month intervals were excluded from the evaluation. To determine the most effective independent predictor, the factors were evaluated by univariate and multivariate logistic regression analysis. The comparisons were made at two distinct time points referred to as “1st” and “5th” using the Mann–Whitney U test. The median values along with the ranges have been provided to delineate the spread of the data in each group. Receiver operating characteristics (ROC) analysis was used to determine the AMH and AFC cut-off levels for predicting CIA. To establish the optimal cut-off points, we aimed to achieve the best balance between sensitivity and specificity for our classifier. The ideal cut-off was determined using Youden’s J index, a criterion that maximizes the combined performance of sensitivity and specificity [24]. *p* < 0.05 was considered statistically significant. All statistical analyses were performed using the SPSS 22.0 software suite (IBM Inc., Chicago, IL, USA).

## 3. Results

The median age of the remaining 71 patients was 38 (range: 23–45), and the mean follow-up period was 37 months (range: 20–51). The clinicopathological characteristics of all patients are shown in Table 1.

Among the patients aged above 35 years, a notably higher proportion experienced chemotherapy-induced amenorrhea (CIA) compared to their counterparts below 35 years—75% vs. 39%. Additionally, the older age group was less frequently treated with an LHRH agonist, with only 30% receiving the treatment compared to 61% in the younger group.

A year following the start of chemotherapy, there was a significant decrease in both AMH levels and AFC. However, MOV remained unaffected. When assessing the correlation between age and reproductive markers, age exhibited a negative relationship with both the initial and subsequent AFC and AMH levels. Specifically, the first and fifth AFC levels both showed a declining trend with increasing age, as did the AMH levels. Conversely, MOV levels at these two time points showed no significant association with age.

During the follow-up, only about 40% of patients experienced a resumption of their menstrual cycle.

Delving into the effect of the estrogen receptor (ER) status on outcomes, patients with ER-positive tumors had lower instances of both CIA and menstrual cycle resumption compared to those with ER-negative tumors. Yet, the choice of chemotherapy regimen, whether TC or AC, or the inclusion or exclusion of taxanes did not seem to play a significant role in either CIA development or menstrual cycle recovery.

Lastly, body mass index (BMI) also offered some insights. While there was not a significant difference in CIA occurrence between the two BMI groups (≤30 and >30), patients with a BMI exceeding 30 displayed a noticeable decrease in their AMH levels after the first year of treatment when compared to those with a BMI of 30 or below.

Our results (Table 2) are summarized below.

Age group ≤35 years vs. >35 years:

- AMH levels were significantly higher in participants aged 35 or younger at both the initial and later stages (*p* = 0.001, *p* = 0.002).
- The AFC displayed higher median values in the younger age group at both time points, with a significant difference initially (*p* = 0.007).
- MOV showed no significant difference between the groups at any stage.
- Age group ≤ 40 years vs. >40 years:
- The AMH levels were considerably higher in individuals aged 40 or younger at both the beginning and later periods (*p* < 0.001, *p* = 0.001).
- AFC and MOV demonstrated no significant difference between the groups at any time point.

Before treatment, the AMH and AFC values were assessed among the patients. By the end of the first year, those who had developed chemotherapy-induced amenorrhea (CIA) presented significantly lower AMH values compared to those who did not develop CIA. Specifically:

- The median pre-treatment AMH values were 0.755 for the CIA group vs. 1.520 for the non-CIA group.
- The median pre-treatment AFC values were 4.50 for the CIA group vs. 12 for the non-CIA group.
- The median AMH value at the end of the first year was 0.010 for the CIA group and 0.073 for the non-CIA group.

These differences were statistically significant, as indicated by their respective *p*-values.

Subsequently, a multivariate analysis was performed to understand the factors predicting CIA (Table 3). Based on the univariate analysis, several factors were identified as significant:

- Age (≤35 vs. >35).
- ER positivity.
- Pre-treatment AMH level.
- Pre-treatment AFC.

From these, ER positivity, the pre-treatment AMH level, and pre-treatment AFC emerged as the significant independent parameters that could predict CIA.

In a separate regression analysis aimed at identifying factors predicting the resumption of menstrual cycles post-treatment, ER positivity stood out as the only statistically significant factor. It demonstrated an odds ratio of 0.6, with a 95% confidence interval ranging from 0.018 to 0.249.

**Figure 2:**
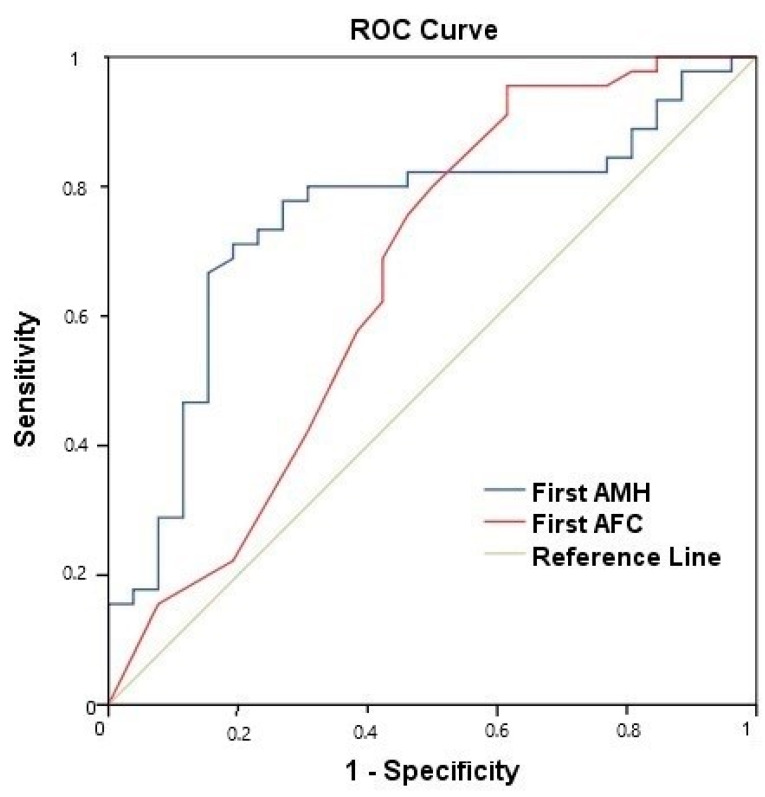
ROC curve analysis of first AMH and AFC levels for predicting CIA. First AMH cut-off value: AMH < 1.475 ng/mL, (Area Under Curve (AUC): 0.746, sensitivity: 85%, specificity 70%, *p* = 0.001). First AFC cut-off value: AFC < 4.5 follicles, (AUC 0.659, sensitivity: 62%, specificity 58%, *p* = 0.02).

It was found that an AMH level of <1.475 ng/mL could predict the development of amenorrhea within 3 years (AMH < 1.475 ng/mL, Area Under Curve (AUC): 0.746, sensitivity: 85%, specificity: 70%, *p* = 0.001; AFC < 4.5 follicles, AUC: 0.659, sensitivity: 62%, specificity: 58%, *p* = 0.02) (Figure 2).

## 4. Discussion

AMH is an indirect parameter for measuring the follicle pool. High levels of AMH affect the quality and number of follicles and provide better fertility rates [10]. In many studies, it was observed that AMH levels and AFC were affected by chemotherapy and decreased significantly with treatment [8,10,17]. In our study, the AMH and AFC values were significantly decreased during follow-up in breast cancer patients who received adjuvant chemotherapy. Pre-chemotherapy AMH levels and AFC showed a statistically significant negative correlation with age, whereas MOV did not. CIA development is an important parameter indicating the ovarian reserve [9]. We found that AMH and AFC values measured before chemotherapy are the earliest parameters to predict CIA development in pre-menopausal women undergoing chemotherapy for breast cancer. In our study, CIA was defined as the discontinuation of menstruation for 6 months after chemotherapy, the same definition used by a previous study [23]. It was determined that an AMH level of <1.475 ng/mL could predict the development of amenorrhea within 3 years (AUC: 0.746, sensitivity: 85%, specificity: 70s%, *p* = 0.001). In addition, there was no difference between the chemotherapy regimens (including or not including taxane) and between the AC and TC regimens in terms of CIA development.

A salient finding from Table 1 pertains to the age of patients and its correlation with chemotherapy-induced amenorrhea (CIA). Age evidently plays a pivotal role in predicting the onset of CIA. Those aged 35 and below displayed a pronounced difference between the CIA and non-CIA groups, with a higher percentage (55%) in the non-CIA category. This difference becomes even more accentuated when we shift our focus to the age bracket of 40 years. While 40% of patients in the CIA group were aged 40 or below, a striking 100% of the non-CIA group fell within this age range, emphasizing the significance of age as a determinant factor. The absence of any patients over 40 in the non-CIA group, coupled with a highly significant *p*-value of <0.001, indicates a clear age-related trend. Such findings underscore age as a potential predictor for CIA, suggesting that younger breast cancer patients might be less susceptible to CIA, a crucial insight that can influence therapeutic decisions and patient counseling.

AMH levels and AFCs were shown to be more efficient than age, FSH, and inhibin B levels in predicting ovarian reserve and fertilization [25]. In our study, while both AMH levels and AFCs were identified as strong predictors of ovarian reserve and fertilization, it is worth emphasizing that the statistical significance of AFC was most pronounced at the first measurement. This suggests that the initial AFC measurement might be a particularly reliable indicator in the early stages of evaluation. Thus, AMH levels and early AFC measurements, in this context, proved to be more effective predictors than age, FSH, and inhibin B levels. Wenners A. et al.’s study with 51 breast cancer patients revealed that AFC and AMH values decreased significantly with advanced age, and AFC, AMH, and MOV values decreased significantly after adjuvant chemotherapy [26]. In another study in which AFCs and AMH levels were evaluated, the AMH value of those who had more than four follicles was >1.2 ng/mL and that of those who had less than four follicles was found to be ≤1.2 ng/mL as a result of induction chemotherapy. In a study of fifty-two patients, it was reported that the values after 6 months of treatment, in which both AMH and AFC values predicted ovarian reserve, were higher in the patients whose menstrual cycle had returned [27]. According to a retrospective analysis conducted with 107 patients in 2020, age was found to be an important factor in patients with breast cancer who developed amenorrhea due to chemotherapy [28]. The fact that the majority of patients were over the age of 40 (78%) should be considered as an important factor affecting this result. In our study, the number of patients over the age of 40 was 38%.

In our study, chemotherapies suitable for current adjuvant treatments were given. In addition, the effects of the taxane-containing chemotherapy regimen and taxane-free chemotherapy regimens on CIA were investigated in a subgroup analysis; AC and TC regimens were compared in terms of the development of CIA and no difference was found. The AMH, AFC, and MOV values of the patients were compared in terms of CIA at the baseline and follow-up. Similar to our study, Anderson et al. revealed that long-term ovarian function after chemotherapy could be predicted by pre-treatment serum AMH concentration. This may also be of value in counseling the patient regarding the need for fertility preservation procedures before commencing therapy [29]. In our study, AMH levels and AFCs were found to be the most effective and the earliest independent parameters for the prediction of CIA. Xue C et al. reported, retrospectively, that CIA could be predicted with 74% sensitivity and 81.4% specificity for an AMH level of <0.965 ng/mL in 120 pre-menopausal patients [30]. D’Avila et al. reported that an AMH level of <3.32 ng/mL and AFC of <13 follicles were correlated with a significantly higher risk of amenorrhea or oligomenorrhea development after cyclophosphamide treatment [31]. There is no clear consensus in the literature on studies on AMH levels [32]. In our prospective study, CIA development was predicted for the 3 years following SC in patients with an AMH level of <1.475 ng/mL, with 85% sensitivity and 70% specificity (AUC: 0.746, *p* = 0.001). AFC < 4.5 follicles predicted CIA with a lower sensitivity and specificity than the AMH level (AUC: 0.659, sensitivity: 62%, specificity: 58%, *p* = 0.02). In addition, the continuation of fertility may be more likely in patients with high pre-treatment AMH levels. Ovum cryopreservation is the most commonly used method for the prevention of fertility [8,15,17,20]. Although there is limited information in the literature, the rate of healthy infants is 20–30% in ovarian cryopreservation and is dependent on the age and ovarian reserve of the oncology patients [33]. It was reported that LHRH agonists started one week before chemotherapy were safe in both hormone receptor-positive and -negative patients, and it was reported that they had a healthy infant rate of 10–15%. It is safe to use and does not cause time loss [34,35]. According to the results of the current study, if patients have high AMH levels and AFCs before chemotherapy and do not have enough time or have a second surgical indication, the use of LHRH agonists independently of the chemotherapy regimen may be considered in clinical practice. The current prospective study revealed that women receiving adjuvant chemotherapy for breast cancer who had an initial AMH level greater than 1.475 ng/mL could be significantly less likely to develop CIA than those with levels lower than 1.475 ng/mL.

The rate of menstrual cycle resumption after CIA was 40%, similar to another study [27], even though the median follow-up period of our study was longer (37 months vs. 14 months). According to the findings of Petrek et al., the mean time required for the return of menstrual cycles in patients who developed transient amenorrhea is 15 months; therefore, the follow-up period in this study should be considered sufficient for evaluation [36]. According to Sukumvanic et al., a 2–3-year period is sufficient for the return of the menstrual cycle [37].

The AMH values measured at the end of the first year of treatment were found to be significantly lower in those with a BMI of more than 30 in accordance with the literature [38].

This study revealed that measurements of AMH levels and AFCs before adjuvant chemotherapy should be advised to pre-menopausal breast cancer patients who desire to know if their menstrual cycles will continue or not.

### 4.1. Clinical Implications

In a clinical and practical context, this research brings forward valuable insights that could be utilized to enhance the holistic care approach for pre-menopausal women undergoing chemotherapy for breast cancer, particularly pertaining to their reproductive health. The pivotal findings regarding the AMH levels and AFC as early indicators of chemotherapy-induced amenorrhea (CIA) development underpin a potential new paradigm in pre-emptive measures and counseling in fertility preservation. This is especially salient for patients who harbor concerns about their post-chemotherapy fertility or are considering future family planning. Our findings of specific AMH and AFC thresholds associated with CIA risk can be operationalized to create risk profiles for patients prior to chemotherapy initiation, thereby facilitating informed decision-making regarding fertility preservation strategies, such as ovum cryopreservation or the use of LHRH agonists. Moreover, this research offers a valuable contribution to the existing body of knowledge in oncology and reproductive medicine by demonstrating the potential of pre-treatment AMH and AFC values as predictive markers, which could be incorporated into standardized pre-chemotherapy evaluations and management protocols. Amidst the myriad of challenges faced by breast cancer patients, offering transparent, data-driven counsel on potential long-term ovarian impacts of chemotherapy based on our findings not only stands to enhance patient autonomy and satisfaction but also adds a nuanced, patient-centric dimension to oncological care.

### 4.2. Limitations

However, there are several limitations to this study. One limitation is the small sample size. The AMH and AFC values could predict ovarian reserve but we need to have longer follow-ups. Another limitation is the varying definitions of CIA among different studies. In the meta-analysis by Wang et al., which examined 68 studies, 13 studies used 3 months as the minimal duration of amenorrhea, 19 studies used 6 months, and 20 studies used 12 months. Moreover, 17 studies did not explicitly state the definition of CIA [39]. Another limitation that we should mention is that the patients were not treated with platinum-based chemotherapy. Therefore, we could not investigate how platinum-based regimens affected amenorrhea in our study.

## 5. Conclusions

Pre-chemotherapy AMH levels and AFCs showed a statistically significant negative correlation with age, whereas MOV did not. In conclusion, AMH and AFC values measured before chemotherapy are the earliest parameters for predicting CIA development in pre-menopausal women undergoing chemotherapy for breast cancer. There was no difference between the chemotherapy regimens (including or not including taxane) or between the AC and TC regimens in terms of CIA development.

## Figures and Tables

**Table 1:** Patient’s characteristics compared with CIA (n = 45) or without CIA (n = 26).

	CIA	Non-CIA	*p*-Value
	n (%)	n (%)	
**Age groups**			
Age ≤ 35 years	9 (20%)	14 (55%)	**0.003**
Age > 35 years	36 (80%)	12 (45%)	
**Age (median–min–max)**	41 (24–45)	35 (23–40)	
**Age groups**			
Age ≤ 40 years	18 (40%)	26 (55%)	**<0.001**
Age > 40 years	27 (60%)	0 (0%)	
**Body mass index**			
BMI≤30	38 (84%)	25 (96%)	0.13
BMI>30	7 (16%)	1 (4%)	
**ER receptor**			
Negative	14 (31%)	2 (8%)	**0.02**
Positive	31 (69%)	24 (92%)	
**PR receptor**			
Negative	16 (36%)	4 (15%)	0.06
Positive	29 (64%)	22 (84%)	
**Histologic grade**			
Grade 1	-	2 (7.7%)	0.16
Grade 2	17 (37.8%)	9 (34.6%)	
Grade 3	28 (63.4%)	15 (57.7%)	
**Molecular subtype**			
Luminal A	8 (18%)	4 (16%)	0.15
Luminal B	22 (50%)	20 (77%)	
HER-2	8 (18%)	1 (3.5%)	
TNBC	6 (14%)	1 (3.5%)	
**pT stage**			
pT1	10 (22%)	7 (27%)	0.47
pT2	24 (53%)	16 (61.5%)	
pT3	11 (25%)	3 (12.5%)	
**Pathologic stage (TNM 8th)**			
Stage 1	29 (64.4%)	18 (69.2%)	0.19
Stage 2	8 (17.8%)	7 (26.9%)	
Stage 3	8 (17.8%)	1 (3.8%)	
**Type of axillary surgery**			
ALND	24 (54%)	10 (38.5%)	0.26
SLNB	21 (46%)	16 (61.5%)	
**Radiotherapy**			
Yes	34 (89.5%)	18 (95%)	0.71
No	4 (10.5%)	1 (5%)	
**Taxane-based regimen**			
Taxane	21 (47%)	10 (38.5%)	
Without taxane	24 (53%)	16 (61.5%)	0.50
**Type of ChT**			
AC	15 (65%)	8 (61.5%)	0.82
TC	8 (35%)	5 (38.5%)	
**ChT cycle number**			
>6	26 (58%)	5 (42%)	0.38
≤6	19 (42%)	21 (58%)	

Chemotherapy-induced amenorrhea (CIA), adriamycin-cyclophosphamide (AC), docetaxel-cyclophosphamide (TC), ER (estrogen receptor), and PR (progesterone receptor). BMI (body mass index), ALND (axillary lymph node dissection), TNBC (triple negative breast cancer), RT (radiotherapy), and Cht: chemotherapy.

**Table 2:** Differential impact of age on ovarian reserve markers at initial and final assessments.

	Age ≤ 35 Years (n = 23)	Age > 35 Years (n = 48)	*p* Score
**AMH (** **1st)**	1.84 (0.03–5.50)	0.71 (0.01–6.20)	**0.001**
**AMH (5th)**	0.27 (0.01–4.30)	0.01 (0.01–4.76)	**0.002**
**AFC (** **1st)**	7 (0–24)	3 (0–21)	**0.007**
**AFC (5th)**	1 (0–8)	0 (0–8)	0.05
**MOV (** **1st)**	6.46 (0.93–42.0)	6.27 (1.01–27.6)	0.26
**MOV (5th)**	3.47 (0.58–15.3)	3.73 (1.10–15.6)	0.80
	**Age ≤ 40 years** **(n = 44)**	**Age > 40 years** **(n = 27)**	***p* score**
**AMH (1st)**	1.52 (0.03–6.20)	0.36 (0.01–3.91)	**<0.001**
**AMH (5th)**	0.06 (0.01–4.76)	0.01 (0.01–0.23)	**0.001**
**AFC (1st)**	4.5 (0–24)	3 (0–13)	0.09
**AFC (5th)**	1 (0–8)	0 (0–5)	0.08
**MOV (1st)**	6.24(0. 39–42.0)	6.33 (2.08–23.3)	0.60
**MOV (5th)**	3.78 (0.58–15.6)	3.20 (1.10–10.1)	0.20

AFC: antral follicle number, AMH: antral Mullerian hormone, and MOV: median ovarian volume.

**Table 3:** Multivariate analysis with logistic regression for chemotherapy-induced amenorrhea.

	Univariate Analysis					Multivariate Analysis				
	OR	95% CI			*p*-Value	OR	95% CI			*p*-Value
**ER −/+**	0.19	0.038	**-**	0.891	**0.03**	0.96	0.94	-	0.98	**0.002**
**HER2 −/+**	0.80	0.435	-	2.936	1.13					
**ALND −/+**	1.75	0.653	-	4.702	0.26					
**AMH(pre-chemo)**	0.64	0.454	-	0.914	**0.01**	0.58	0.353	-	0.952	**0.03**
**AFC (pre-chemo)**	0.87	0.780	-	0.961	**0.007**	0.75	0.574	-	0.958	**0.02**
**MOV (pre-chemo)**	1.00	0.987	-	1.021	0.68					
**Taxane (+/−)**	0.71	0.267	-	1.910	0.50					
**TC/AC**	0.85	0.209	-	3.491	0.82					
**Age ≤35/>35**	4.66	1.613	-	13.498	**0.004**					
**BMI ≤25/>25**	2.57	0.929	-	7.118	0.06					

Chemotherapy-induced amenorrhea (CIA), adriamycin-cyclophosphamide (AC), docetaxel-cyclophosphamide (TC), ER (estrogen receptor), and PR (progesterone receptor). BMI (body mass index), ALND (axillary lymph node dissection), TNBC (triple negative breast cancer), RT (radiotherapy), postCht: post-chemotherapy, AFC: antral follicle number, AMH: antral Mullerian hormone, and MOV: median ovarian volume.

## Data Availability

The datasets generated during and/or analyzed during the current study are available from the corresponding authors upon reasonable request.

## References

[1] Ünal I.Ö., Ordu C. (2023). Decoding Caregiver Burden in Cancer: Role of Emotional Health, Rumination, and Coping Mechanisms. Healthcare.

[2] Ozonder Unal I., Ordu C. (2023). Alexithymia, Self-Compassion, Emotional Resilience, and Cognitive Emotion Regulation: Charting the Emotional Journey of Cancer Patients. Curr. Oncol..

[3] Sung H., Ferlay J., Siegel R.L., Laversanne M., Soerjomataram I., Jemal A., Bray F. (2021). Global Cancer Statistics 2020: GLOBOCAN Estimates of Incidence and Mortality Worldwide for 36 Cancers in 185 Countries. CA Cancer J. Clin..

[4] Ünal Ç., Özmen T., Ordu Ç., Pilanci K.N., İ lgün A.S., Gökmen E., Almuradova E., Özdoğan M., Güler N., Uras C. (2023). Survival results according to Oncotype Dx recurrence score in patients with hormone receptor positive HER-2 negative early-stage breast cancer: First multicenter Oncotype Dx recurrence score survival data of Turkey. Front. Oncol..

[5] Rosenberg S.M., Newman L.A., Partridge A.H. (2015). Breast Cancer in Young Women: Rare Disease or Public Health Problem?. JAMA Oncol..

[6] Ozmen V., Ozmen T., Dogru V. (2019). Breast Cancer in Turkey; An Analysis of 20.000 Patients with Breast Cancer. Eur. J. Breast Health.

[7] Kim S.E., Kim W.-J., Choi D., Lee D.-Y. (2023). Comparison of goserelin and leuprorelin for ovarian protection during chemotherapy in young patients with breast cancer. Breast Cancer Res. Treat..

[8] Fabiani C., Guarino A., Meneghini C., Licata E., Paciotti G., Miriello D., Schiavi M.C., Spina V., Corno R., Gallo M. (2022). Oocyte Quality Assessment in Breast Cancer: Implications for Fertility Preservation. Cancers.

[9] Bedoschi G., Navarro P.A., Oktay K. (2016). Chemotherapy-induced damage to ovary: Mechanisms and clinical impact. Futur. Oncol..

[10] Wu M., Zhu Q., Huang Y., Tang W., Dai J., Guo Y., Xiong J., Zhang J., Zhou S., Fu F. (2023). Ovarian reserve in reproductive-aged patients with cancer before gonadotoxic treatment: A systematic review and meta-analysis. Hum. Reprod. Open.

[11] Ünal Ç., Tunçer G., Çopur B., Pilanci K.N., Okutur K.S., Yararbaş K., Alan Ö., Sakin A., Simsek M., Ünal İ.Ö. (2023). Clinical and inflammation marker features of cancer patients with COVID-19: Data of Istanbul, Turkey multicenter cancer patients (2020–2022). Curr. Med. Res. Opin..

[12] Kalelioglu T., Karamustafalioglu N., Emul M., Celikel G., Ozonder I., Kara A., Kilic C., Yalcin S., Celik E., Kilic U. (2023). Detecting biomarkers associated with antipsychotic-induced extrapyramidal syndromes by using machine learning techniques. J. Psychiatr. Res..

[13] Bedenk J., Vrtačnik-Bokal E., Virant-Klun I. (2020). The role of anti-Müllerian hormone (AMH) in ovarian disease and infertility. J. Assist. Reprod. Genet..

[14] Amir E., Freedman O., Allen L., Colgan T., Clemons M. (2010). Defining ovarian failure in amenorrheic young breast cancer patients. Breast.

[15] Arecco L., Blondeaux E., Bruzzone M., Ceppi M., Latocca M.M., Marrocco C., Boutros A., Spagnolo F., Razeti M.G., Favero D. (2022). Safety of fertility preservation techniques before and after anticancer treatments in young women with breast cancer: A systematic review and meta-analysis. Hum. Reprod..

[16] Song Y., Liu H. (2021). A review on the relationship between anti-mullerian hormone and fertility in treating young breast cancer patients. BMC Women’s Health.

[17] Furlanetto J., Marmé F., Seiler S., Thode C., Untch M., Schmatloch S., Schneeweiss A., Bassy M., Fasching P.A., Strik D. (2021). Chemotherapy-induced ovarian failure in young women with early breast cancer: Prospective analysis of four randomised neoadjuvant/adjuvant breast cancer trials. Eur. J. Cancer.

[18] Zavos A., Valachis A. (2016). Risk of chemotherapy-induced amenorrhea in patients with breast cancer: A systematic review and meta-analysis. Acta Oncol..

[19] Mir M.A., Mir A.Y. (2023). Current Treatment Approaches to Breast Cancer. Therapeutic Potential of Cell Cycle Kinases in Breast Cancer.

[20] Gracia C.R., Jeruss J.S. (2013). Lives in the Balance: Women With Cancer and the Right to Fertility Care. J. Clin. Oncol..

[21] Giovannopoulou E., Karakasi M.-V., Kouroupi M., Giatromanolaki A., Tsikouras P., Pavlidis P. (2023). Safety and efficacy of ovarian tissue autotransplantation: A systematic literature review. Folia Medica.

[22] Çelebi F., Ordu Ç., Ilgün S., Oztürk A., Erdoğan Iyigün Z., Alço G., Duymaz T., Aktepe F., Soybir G., Baysal B. (2020). 2020, The Effect of Systemic Chemotherapy on Ovarian Function: A Prospective Clinical Trial. Eur. J. Breast Health.

[23] Park I.H., Han H.S., Lee H., Lee K.S., Kang H.S., Lee S., Kim S.W., Jung S., Ro J. (2012). Resumption or persistence of menstruation after cytotoxic chemotherapy is a prognostic factor for poor disease-free survival in premenopausal patients with early breast cancer. Ann. Oncol..

[24] Lu J., Karwoski A., Abdulrahman L., Chaparala S., Chaudhary M., Nagarsheth K. (2023). Neutrophil-to-Lymphocyte Ratio as a Predictor of Mortality for COVID-19-Related Acute Respiratory Distress Syndrome (ARDS) Patients Requiring Extracorporeal Membrane Oxygenation Therapy. Cureus.

[25] Peluso C., de Oliveira R., Laporta G.Z., Christofolini D.M., Fonseca F.L.A., Laganà A.S., Barbosa C.P., Bianco B. (2021). Are ovarian reserve tests reliable in predicting ovarian response? Results from a prospective, cross-sectional, single-center analysis. Gynecol. Endocrinol..

[26] Wenners A., Grambach J., Koss J., Maass N., Jonat W., Schmutzler A., Mundhenke C. (2017). Reduced ovarian reserve in young early breast cancer patients: Preliminary data from a prospective cohort trial. BMC Cancer.

[27] D’Avila A.M., Capp E., von Eye Corleta H. (2017). Antral Follicles Count and Anti-Müllerian Hormone Levels after Gonadotoxic Chemotherapy in Patients with Breast Cancer: Cohort Study. Rev. Bras. Ginecol. Obstet..

[28] Turnbull A.K., Patel S., Martinez-Perez C., Rigg A., Oikonomidou O. (2021). Risk of chemotherapy-related amenorrhoea (CRA) in premenopausal women undergoing chemotherapy for early stage breast cancer. Breast Cancer Res. Treat..

[29] Anderson R.A., Themmen AP N., Qahtani A.A., Groome N.P., Cameron D.A. (2006). The effects of chemotherapy and long-term gonadotrophin suppression on the ovarian reserve in premenopausal women with breast cancer. Hum. Reprod..

[30] Xue C., Wei W., Sun P., Zheng W., Diao X., Xu F., Huang J., An X., Xia W., Hong R. (2019). Pretreatment anti-Mullerian hormone-based nomogram predicts menstruation status after chemotherapy for premenopausal women with hormone receptor-positive early breast cancer. Breast Cancer Res. Treat..

[31] D’Avila Â.M., Biolchi V., Capp E., von Eye Corleta H. (2015). Age, anti-müllerian hormone, antral follicles count to predict amenorrhea or oligomenorrhea after chemotherapy with cyclophosphamide. J. Ovarian Res..

[32] Moolhuijsen L.M.E., Visser J.A. (2020). Anti-Müllerian Hormone and Ovarian Reserve: Update on Assessing Ovarian Function. J. Clin. Endocrinol. Metab..

[33] Garcia-Velasco J.A., Domingo J., Cobo A., Martı´nez M., Carmona L., Pellicer A. (2013). Five years’ experience using oocyte vitrification to preserve fertility for medical and nonmedical indications. Fertil. Steril..

[34] Lambertini M., Moore H.C., Leonard R.C., Loibl S., Munster P., Bruzzone M., Boni L., Unger J.M., Anderson R.A., Mehta K. (2018). Gonadotropin-Releasing Hormone Agonists During Chemotherapy for Preservation of Ovarian Function and Fertility in Premenopausal Patients With Early Breast Cancer: A Systematic Review and Meta-Analysis of Individual Patient–Level Data. J. Clin. Oncol..

[35] Moore H.C., Unger J.M., Phillips K.-A., Boyle F., Hitre E., Porter D., Francis P.A., Goldstein L.J., Gomez H.L., Vallejos C.S. (2015). Goserelin for Ovarian Protection during Breast-Cancer Adjuvant Chemotherapy, POEMS/S0230. N. Engl. J. Med..

[36] Petrek J.A., Naughton M.J., Case L.D., Paskett E.D., Naftalis E.Z., Singletary S.E., Sukumvanich P. (2006). Incidence, time course, and determinants of menstrual bleeding after breast cancer treatment: A prospective study. J. Clin. Oncol..

[37] Sukumvanich P., Case L.D., Van Zee K., Singletary S.E., Paskett E.D., Petrek J.A., Naftalis E., Naughton M.J. (2010). Incidence and time course of bleeding after long-term amenorrhea after breast cancer treatment: A prospective study. Cancer.

[38] Ordu Ç., Pilancı K.N., Alço G., Elbüken F., Köksal Ü.İ., İlgun S., Sarsenov D., Aydın A.E., Öztürk A., İyigün Z. (2018). Prognostic Significance of Adjuvant Chemotherapy Induced Amenorrhea in Luminal A and B Subtypes. Eur. J. Breast Health.

[39] Wang Y., Li Y., Liang J., Zhang N., Yang Q. (2022). Chemotherapy-Induced Amenorrhea and Its Prognostic Significance in Premenopausal Women With Breast Cancer: An Updated Meta-Analysis. Front. Oncol..

